# The effects of environmental variability and forest management on natural forest carbon stock in northwestern Ethiopia

**DOI:** 10.1002/ece3.11476

**Published:** 2024-06-06

**Authors:** Melkamu Kassaye, Yonas Derebe, Wondwossen Kibrie, Fikadu Debebe, Etsegenet Emiru, Bahiru Gedamu, Mulugeta Tamir

**Affiliations:** ^1^ Department of Forestry and Climate Science Injibara University Injibara Ethiopia; ^2^ Department of Natural Resources Management Injibara University Injibara Ethiopia

**Keywords:** carbon stock, climate change, environmental factors, forest management, natural forest

## Abstract

Natural forests are crucial for climate change mitigation and adaptation, but deforestation and degradation challenges highly reduce their value. This study evaluates the potential of natural forest carbon stock and the influence of management interventions on enhancing forest carbon storage capacity. Based on forest area cover, a study was conducted in nine purposely selected forest patches across various forest ecosystems. Data on diameter, height, and environmental variables from various forest management approaches were collected and analyzed with R Ver. 4.1. The findings revealed a substantial difference (*p* .029) in carbon stock between environmental variables and management interventions. The findings revealed a strong connection between environmental variables and the overall pool of carbon stock within forest patches (*p* .029). Carbon stocks were highest in the Moist‐montane forest ecosystem (778.25 ton/ha), moderate slope (1019.5 ton/ha), lower elevation (614.50 ton/ha), southwest‐facing (800.1 ton/ha) and area exclosures (993.2 ton/ha). Accordingly, natural forests, particularly unmanaged parts, are sensitive to anthropogenic stresses, decreasing their ability to efficiently store carbon. As a result, the study highlighted the importance of sustainable forest management, particularly area exclosures and participatory forest management, in increasing forest carbon storage potential.

## Graphical abstract

According to the study, the natural forest in the study area has significant carbon storage, which is essential for reducing climate change's effects and preparing for them. However, forest management practices like area enclosures boost the forest's ability to store carbon, while anthropogenic pressure declines the value of forest resources.

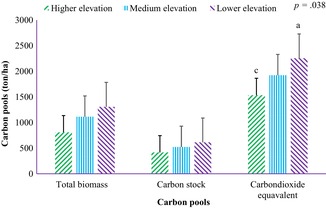

## 1 INTRODUCTION

Natural disasters and climate change are two major global environmental challenges caused mostly by fossil fuels and pollution. This is caused to the overuse and depletion of natural resources, particularly vegetation. Climate change had several consequences, including global warming, glacier melting (frequent floods), more fires, and recurring droughts. This is a universal problem that has received significant attention in research and project intervention (Ostwald & Ravindranath, 2008). Forests have an important role in reducing greenhouse gas emissions through carbon sequestration, as well as maintaining natural balance (Malhi, 2010). There has been limited scientific research on the CO_2_ sequestration capacity of these forest resources in connection to environmental factors and forest management strategies. The majority of carbon pool studies emphasized the importance and role of forest resources in lowering and adjusting to greenhouse gas emissions (Bayley et al., 2021; Havu et al., 2022; Joshi et al., 2021; Piffer et al., 2022). However, understanding the potential amount of carbon stored in the forest with specific environmental variables, as well as demonstrating the role of forest management in enhancing forest carbon store potential, are the first steps toward providing credit and financing for carbon under the international clean development mechanism policy.

The studies by Saatchi et al. (2011), Chazdon et al. (2016), and Mo et al. (2023) highlights the significant carbon storage potential of natural forests, particularly in the Amazon, Asia, and tropical forest ecosystems, for mitigating climate change effects. However, forest management practices like enclosures can enhance carbon storage, while anthropogenic pressure can decline the value of forest resources. Natural regeneration of second‐growth forests offers high carbon sequestration potential, but the study's scope is limited and may require future refinement due to differences in forest area assessments. Because African tropical forests, particularly montane forests, store a significant amount of carbon, it is critical that they be protected and maintained responsibly. The average aboveground living tree biomass carbon (AGC) stock in these forests is 149.4 megagrams per hectare, comparable to lowland African forests. The research emphasizes for the forest's carbon pool to increase, sustainable management is required. Nevertheless, as the study relied on remote‐sensing techniques, it is difficult to precisely and thoroughly estimate the carbon stocks in these forests (Cuni‐Sanchez et al., 2021; Lewis et al., 2009).

There is a large amount of aboveground (biomass) and belowground (SOC) in various regions of Ethiopia's forest ecosystems, including natural forests in several churches, southern, northwestern, and Afromontane forest ecosystems. The carbon stock of Ethiopian forests is significantly influenced by altitude, slope, and topographic aspects. Forests on north‐facing slopes have higher carbon stocks compared to those on south‐facing slopes, attributed to variations in microclimate, soil properties, and vegetation composition. However, the studies recommend the sustainable forest management to enhance the carbon stock potential. However, the studies by Kendie et al. (2021), Chimdessa (2023), and Ahmed and Lemessa (2024) acknowledges that information on the carbon stock efficiency of different forest types in Ethiopia is scarce, and the study does not provide details on the methods used to collect primary and secondary data, which may limit the generalizability of the findings. Therefore, understanding these species‐specific dynamics is crucial for effective forest management and climate change mitigation in Ethiopian forests (Asbeck et al., 2021).

Recent studies conducted in the Awi zone (northwestern Ethiopia) by Gebeyehu et al. (2019) and Sewagegn et al. (2022) proved that there is a huge amount of carbon stock in some church forests, and the natural forests are inaccessible for grazing and human encroachment. However, the forests have experienced significant degradation and deforestation due to agricultural land expansion. Disturbance, elevation, soil pH, and stand structure all have a significant impact on the forest carbon store potential. These studies are limited to church forests and isolated forest patches, and thus lack a broad perspective for policymakers based on a forest ecosystem‐based approach that is sensitive to environmental variability. Therefore, studying representative forest patches and specific environmental factors is crucial for policy formulation and sustainable restoration to enhance forest carbon stock potential for climate change mitigation and adaptation.

As a result, this study focuses on (a) quantifying the variation in biomass and carbon stocks among forest ecosystems and forest patches; (b) assessing the impacts of slope, aspect, and elevation on biomass and carbon stocks; (c) exploring the role of selected forest management interventions for biomass and carbon stock improvement; and (d) determining the capacity of a natural forest ecosystem to store soil organic carbon. This study is an essential and fundamental step toward sustainable forest conservation and restoration for reducing climate change and preparing for it (Kassaye et al., 2022). The study makes a substantial contribution to the creation and implementation of policies and strategies for the conservation of forest ecosystem and their use, primarily in measures for coping with and adapting to climate change. Furthermore, understanding the potential amount of carbon stored in forests is the first step toward providing credit and financing for carbon under the international clean development framework policy. International commitments and national initiatives, such as the clean development mechanism, REDD+, and UNFCC, have an intervention action that includes incentives based on the amount of carbon stored in the forest and its geographic distribution, as well as national initiatives by nations to fulfill the commitments (IPCC, 2006).

## 2 MATERIALS AND METHODS

### 2.1 Study area description

**FIGURE 1:**
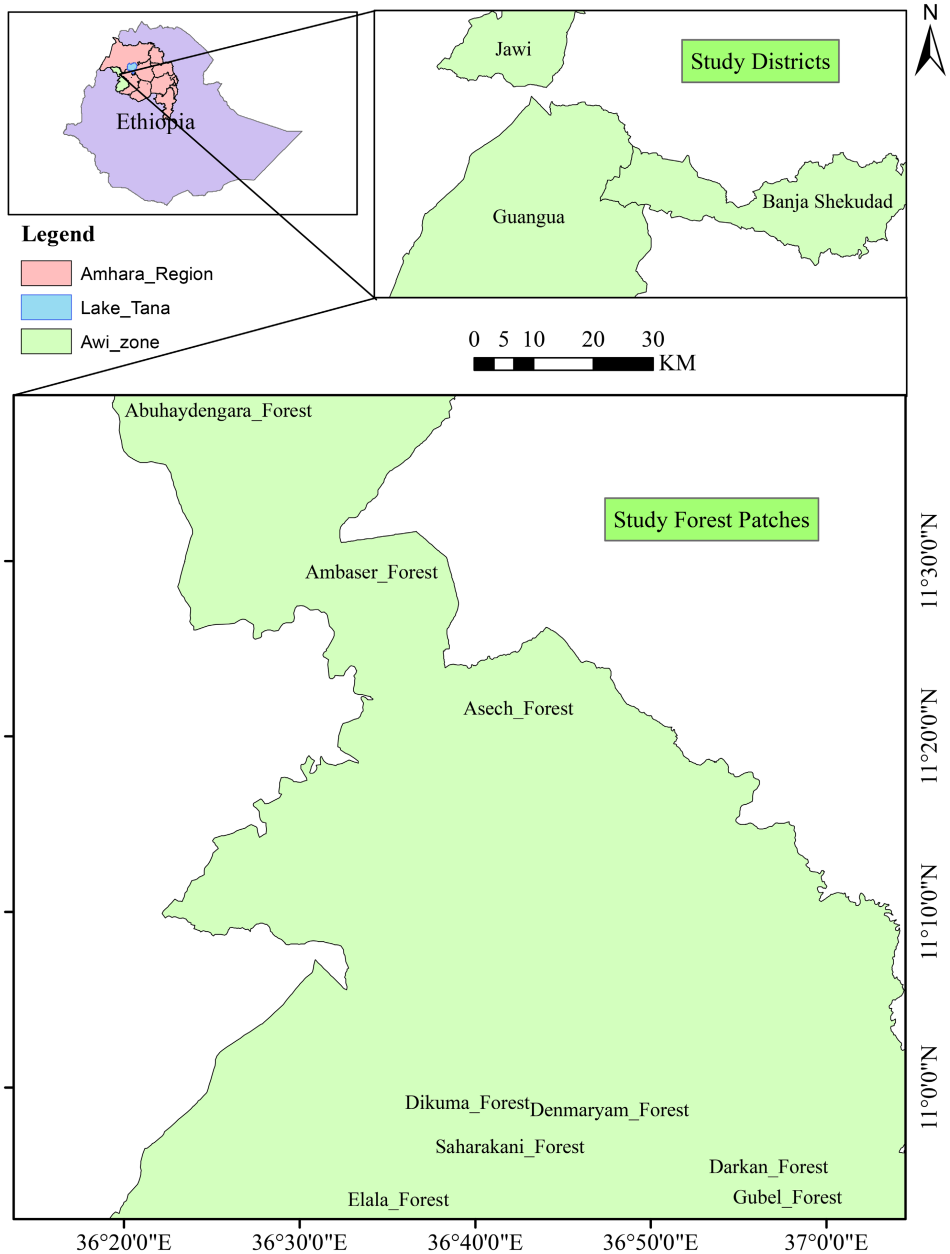
Map of the study area.

The investigation took place in northwestern Ethiopia at latitudes 11°10′85″ N and longitudes 36°39′60″ and 36′57″ E. Its elevation ranges from 600 to 3500 m.a.sl., with an average annual rainfall of 1750 mm and temperatures ranging from 17 to 27°C. The research area is topographically quite flat and productive (Kassaye et al., 2023) (Figure 1).

Awi zone have Woina‐Dega (equivalent to mid‐altitude) (72%), followed by Dega (equivalent to highland) (17%), and Kolla (equivalent to lowland agroclimatic zone) (11%). The study area ranges from 700 to 3200 a. sl. in altitude, and it is the area with the best annual rainfall distribution (800 to 2700 mm/year) in the area. The temperature in the area ranges between 15 and 24°C. Temperatures in the area range from 15 to 24°C (Mekasha et al., 2022).

From the total area of the zone (8,935,520 ha) of land, 297,133 ha (33.25%) are used for farm practices. However, most of the area in the Awi zone (34.02%) (of 76,554 ha of plantation and 277,842 ha of natural forest) is covered with forest. Rangeland and grazing land cover 24.3% (217,138 ha) of the total area, and other land uses like infrastructure and settlement cover 8.38% (74,853 ha) of the of the area (Table 1).

**TABLE 1:** The forest cover of study areas (Awi ZOne Agricultural office, 2021).

Agroclimatic zones	Total area (ha)	Area of natural forest (ha)	Cover (%)	Area of plantation (ha)	Cover (%)	Total cover (%)
Highland	47,915.8	2679	5.6	18,752.7	39.13	44.7
Mid‐altitude	107,195	21,956	20.5	8976.4	8.4	28.8
Lowland	332,671	162,775	48.9	2663.5	0.8	49.7

### 2.2 Study forest description and sampling techniques

First, three agroclimatic zones—Highland (2300–3200), Mid‐altitude (1500–2200), and Lowland (500–1500) m.a.sl.—were identified as the research area (Azene, 2007; Gorfu & Ahmed, 2011). In each agroclimatic zone parallel forest ecosystem was selected. The studies by Kelbessa and Girma (2011) and Asefa et al. (2020) classified Ethiopian forests into seven ecosystems (Afroalpine, Sub‐Afroalpine, moist montane, dry montane, montane grassland, *Combretum‐Terminalia* = broad‐leaved deciduous woodland, and *Acacia‐Commiphora* woodland) based on altitude, climatic conditions, and species composition and characteristics. The study forests in northwestern Ethiopia (Awi zone) have been segmented into three forest ecosystems according to the above classification. As a result, three forest ecosystems were selected for study: moist‐montane (highland), dry‐evergreen‐montane (mid‐altitude), and broad‐lived deciduous (lowland). Three forest patches for each forest ecosystem were selected (one open/not managed, one for PFM, and one for area exclosures). Secondary data for forest patches under management were obtained from the Awi zone Forest and Environmental Protection Department. These data were validated by field observations (observing the forest prior to the study, checking the forest guards, and interviewing local residents). The report provided information on the years since management intervention as well as the area of each forest patch (Table 2). Thus, a total of nine forest patches were selected for this study. These forest patches, under different management interventions and forest ecosystems, were selected purposefully based on their area coverage. That means the forest patch with the highest forest cover (ha) was selected for each forest ecosystem.

**TABLE 2:** Selected forest patches for this study (Awi ZOne Agricultural office, 2021).

Agroclimatic zone^a^	Forest ecosystems^b^	Forest patches^c^ (local name)	Area (ha)	Time since intervention	Current management interventions	No. of quadrates taken
Highland (2300–3200 m. a.sl.).	Moist‐montane forest ecosystem	Gubel	140.9	1999	Area exclosures^d^	10
		Darkan	160.7		Open forest^e^	8
		Saharakani	379	2000	PFM^f^	5
Mid‐altitude (1500–2300 m. a. sl.)	Dry‐evergreen montane forest	Den Maryam	574		Open forest	10
		Elala	574	2001	PFM	13
		Dikuma	502	1998	Area exclosures	13
Lowland (500–1500 m. a. sl.)	Broad‐leaved deciduous forest	Asech	8.4		Open forest	5
		Ambaser	11.93	2005	Area exclosures	3
		Abuhay Dengara	14.1	2006	PFM	5
Total plots						72

*Note*: Working definition for this study (from Table 2).

^a^ Agroclimatic zone is a land unit accurately represented in terms of major climate conditions and growing periods, making it conducive for specific vegetation and agricultural crops.

^b^ Forest ecosystem is complex and interconnected community of living organisms (both plants and animals) that inhabit a variety of forest patches.

^c^ Forest patch is the fragmented forests after long and intensive encroachment within forest ecosystem.

^d^ Area exclosures is the forest restoration strategy by excluding human and grazing interferences.

^e^ PFM is the participatory forest management at which local community engage in the forest management with the support of NGOs and the government.

^f^ Open Forest is the forest communally accessed by all communities without any restriction.

### 2.3 Data collection

Following forest patch selection, the first quadrate, measuring 20 m by 20 m square, was randomly placed 100 m from the forest edge. The second quadrate was then established sequentially at a distance of 400 m from the transect lines and 250 m between them as well. The quadrates and transect lines were placed using GPS throughout the elevation gradient. This idea is based Kent's (2012) vegetation description and analysis approaches for the majority of tropical and subtropical forest ecosystems. The height and diameter of trees with a diameter of 5 cm or more were measured in each quadrate. A total of 72 square quadrates were examined throughout nine forest patches. Height in hypsometer, diameter in diameter tape, and environmental patterns with GPS were collected in these forest patches for evaluation of forest biomass and carbon stock potential. Site parameters such as slope class (gentle slope = 1%–10%, mid‐slope = 10.1%–20%, and upper slope = 20.1%–30%), aspect angle data were collected at each quadrate and then classified as Northeast (NE), Northwest (NW), Southeast (SE), and Southwest (SW), and elevation were classified for individual forest patches and collected with GPS from each quadrate (Vásquez‐Grandón et al., 2018).

### 2.4 Data analysis

First, data were collected at each quadrate (0.04 ha) and prepared on a on a sheet before being encoded in the Excel spreadsheet. The factors (independent variables)—forest ecosystems, forest patches, slope, elevation, aspect, and forest management intervention—were then arranged, along with the dependent variables (height and diameter). The aboveground, belowground, total biomass, carbon stock, SOC, and CO_2_ sequestration potential were then computed using Microsoft Excel. The data was then compiled and prepared for inferential and descriptive analysis of the dependent variable in respect to the factors. The aboveground biomass of woody species with DBH ≥5 cm was calculated using allometric equation of Chave et al. (2014).

(1) $$\mathrm{AGB}=0.0673\;{\left(\mathrm{WD}*H*{\mathrm{DBH}}^{2}\right)}^{0.976}$$

where; AGB, aboveground biomass in KG; WD, wood density in kg/m^3^; *H*, height in meter; DBH, diameter at breast height in cm.

This allometric equation was applied for this study because it is widely used and most appropriate in tropical African natural forest trees (Henry, 2010; IPCC, 2006; Nizami, 2010; Toru & Kibret, 2019). Many studies with biomass and carbon estimation of forest and other agricultural land use in tropical Africa, specifically Ethiopia, use this biomass allometric question (Bazezew et al., 2015a; Dibaba et al., 2019; Gedefaw et al., 2013; Kendie et al., 2021; Meragiaw et al., 2021; Siraj, 2019; Solomon et al., 2017).

The wood‐specific density was taken from (Ethiopia's Forest Reference Level Submission to the UNFCCC, 2016) guideline. To simplify the process for estimating belowground biomass, it is recommended that the root‐to‐shoot ratio value of 1:5 is used; that is, to estimate belowground biomass as 20% of aboveground tree biomass (Bhishma et al., 2010) and (Bazezew et al., 2015a).

(2) BGB=0.2*AGB

where: BGB: belowground biomass in kg;

(3) TB=AGB+BGB

(4) CS=TB*0.47

(5) $${\mathrm{CO}}_{2}\mathrm{Seq}=\mathrm{CS}*3.67$$

where; TB, total biomass in Kg; CS, carbon stock (IPCC, 2006).

### 2.5 Soil sample collection and analysis

The five (1 × 1 m) sup‐quadrates at the four corners and in the center were established. The soil samples were then collected at a depth of 20 cm using an augur and composited in the primary quadrate level, followed by three elevations at each forest patch; a total of 27 soil samples were taken to Injibara University soil laboratory for organic carbon analysis. The organic carbon which makes up 58% of soil organic matter, is determined using the modified Walkely–Black method and colorimetric method. These methods involve wet oxidation of organic carbon in an acid dichromate solution, followed by back titration with ferrous ammonium sulphate or photometric determination of Cr^3^+ (Sortsu & Bekele, 2000). Soil bulk density were collected and analyzed using core Method which is utilized when coarse fragments (particles larger than 2 mm in diameter) account for less than 25% of the total volume. A double‐cylinder, drop‐hammer sampler with a core is intended to extract a cylindrical core of soil. The sampling head has an inner cylinder that is pressed into the soil using a drop hammer. The inner cylinder containing an undisturbed soil core is then removed and trimmed to the end using a knife, resulting in a core whose volume can be computed using its length and diameter. The weight of this soil core is then determined after it has been dried in an oven at 105°C for approximately 18–24 h (Sortsu & Bekele, 2000). Then, organic carbon was converted to Soil organic carbon with:

(6) SOC=BD*D*%OC*100

where SOC, Soil Organic Carbon (ton/ha), BD, Bulk Density (g/cm^3^), D, soil depth (cm), OC (%), Carbon concentration in the soil under the forest, 1000 is the conversion factor from g/cm^2^ to ton/ha (Hagos et al., 2021).

Then inferential and descriptive statistics were applied with R Ver.4.1, via carbon as an overall pool and environmental factors, forest ecosystems, forest patches, and forest management interventions.

## 3 RESULTS

### 3.1 Carbon stock across the forest ecosystems and forest patches

Aboveground biomass, belowground biomass, carbon stock, and CO_2_ sequestration potential differed significantly (*p* .001) across forest ecosystems and forest patches (Table A4). Moist‐montane forest ecosystems had the highest carbon stock (778.25 ton/ha and 2856.18 ton/ha CO_2_ sequestration potential), followed by dry‐evergreen‐montane forest ecosystems (471.74 ton/ha carbon stock and 1731.33 ton/ha CO_2_ sequestration potential). The Saharakani forest patch had the highest carbon stock and CO_2_ sequestration potential (1054.59 ton/ha carbon stock and 3870.34 ton/ha CO_2_ sequestration potential), followed by the “Gubel” forest patch (829.88 ton/ha carbon stock and 3045.68 ton/ha CO_2_ equivalent) and the Elala forest patch (648.27 ton/ha carbon stock and 2379 ton/ha CO_2_ equivalent) (Tables 3 and A2).

**TABLE 3:** Woody species biomass and carbon stock (ton ha^−1^) (** shows significant difference at .01 level of significance; the values with a similar letter are insignificant, according to mean separation).

Forest ecosystems	Forest patches	AGB	BGB	TB	Carbon stock	CO_2_ seq
Moist‐montane Forest	Darkan	798.37	159.67	958.05^d^	450.28^d^	1652.53^d^
	Gubel	1471.4	294.29	1765.71^b^	829.88^b^	3045.68^b^
	Saharakani	1869.8	373.97	2243.81^a^	1054.59^b^	3870.34^b^
Dry‐evergreen‐montane Forest	Den Maryam	508.53	101.71	610.23^e^	286.81^e^	1052.59^e^
	Dikuma	851.35	170.27	1021.63^d^	480.16^d^	1762.20^d^
	Elala	1149.4	229.88	1379.30^c^	648.27^c^	2379.16^c^
Broad‐leaved‐deciduous Forest	Asech	690.66	138.13	828.79^d^	389.53^d^	1429.58^d^
	Ambaser	136.32	27.26	163.59^f^	76.89^f^	282.18^f^
	Abuhay Dengara	698.29	139.66	837.95^d^	393.84^d^	1445.39^d^
	Mean	908.25	181.65	1089.90**	512.25**	1879.96**

### 3.2 Effects of environmental patterns on carbon stock

There was a significant difference (*p* .003) between aboveground biomass, belowground biomass, carbon stock, and CO_2_ sequestration potential across the slope gradients. The gentle slope gradient had the highest carbon stock (1019.5 ton/ha) and CO_2_ equivalent (3741.6‐ton/ha), followed by the steep slope (619.7 ton/ha carbon stock and 2274.4 ton/ha CO_2_ equivalent) and medium slope (472.2 ton/ha carbon stock and 1732.8 ton/ha CO_2_ sequestration potential) (Tables 4 and A3).

**TABLE 4:** Carbon stock (ton ha^−1^) across various forest patches and slope patterns (** shows significant difference at .01 level of significance).

Slope	Forest patches	Mid‐altitude			Highland			Lowland			Mean	Significance (.05)
		Den Maryam	Elala	Dikuma	Gubel	Darkan	Saharakani	Asech	Ambaser	Abuhay Dengara		
Gentle	TB	709.1	1601	1419.4	3548.6	1169.5	8785.5	915.6	273.3	1100.4	2169.1	*p* < .003**
	C stock	333.3	752.5	667.1	1667.8	549.7	4129.2	430.3	128.4	517.2	1019.5	
	CO_2_ seq	1223.1	2761	2448.4	6120.9	2017.3	15,154.0	1579.3	471.4	1898.0	3741.6	
Medium	TB	1271.5	689.6	2117.5	2153.5	258.2	787.6	446.6	149.6	1167.5	1004.6	
	C stock	597.6	324.1	995.2	1012.1	121.3	370.2	209.9	70.3	548.7	472.2	
	Co_2_ seq	2193.2	1189	3652.5	3714.5	445.3	1358.5	770.3	258.0	2013.8	1732.8	
Steep	TB	561.9	578.1	2346.9	3698.9	2149.8	2006.7	255.4	157.4	112.2	1318.6	
	C stock	264.1	271.7	1103.1	1738.5	1010.4	943.1	120.0	74.0	52.7	619.7	
	CO_2_ seq	969.2	997.1	4048.2	6380.2	3708.2	3461.3	440.6	271.6	193.6	2274.4	
Mean		902.5	1018	2088.7	3337.2	1270.0	4110.7	574.2	206.0	844.9	1594.7	
Significance (.05)		*p* < .001**										

Carbon pools differed significantly (p 0.04) across aspect gradients. As overall pools, southwest‐facing had the highest carbon stock (800.1 ton/ha) followed by west‐facing (774.2 ton/ha) and southeast‐facing (748.3 ton/ha) (Table 5).

**TABLE 5:** Carbon stock in aspect gradient (different superscript letters show the differnces between means and similar superscript lettes shows no different between means based on mean separation; *shows significant difference at .05 level of significance).

Aspects	Total biomass (ton/ha)	Carbon stock (ton/ha)	CO_2_ sequestration (ton/ha)
North	1153.4^cd^	542.1^cd^	1989.4^cd^
South	1647.3^a^	774.2^a^	2841.4^a^
East	1439.2^b^	674.4^b^	2482.3^b^
West	1361.4^bc^	639.9^bc^	2348.3^bc^
North East	1286.2^c^	604.5^c^	2218.5^c^
North West	1020.5^d^	479.7^d^	1760.3^d^
South East	1592.2^b^	748.3^b^	2746.4^b^
South West	1702.3^a^	800.1^a^	2936.3^a^
Mean	1400.3125*	657.9*	2415.363*

**FIGURE 2:**
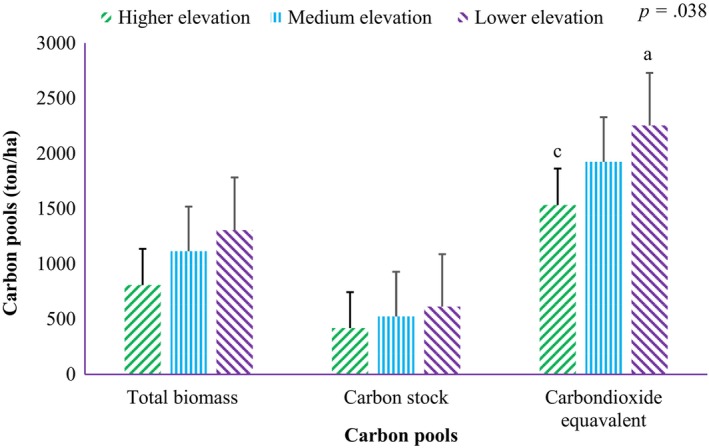
Carbon stock dynamics in elevation pattern (the values with different letters have significant differences according to mean separation).

There was a significant difference (*p* .038) in CO_2_ equivalent among altitudes but not in carbon stock (*p* .57). Lower altitude has the greatest potential for CO_2_ equivalent (2253.5 ton/ha) followed by medium altitude (1923.1 ton/ha) and higher altitude (890.1 ton/ha) (Figure 2, Table A1).

### 3.3 Effects of forest management intervention on carbon stock

Forest carbon pools differed significantly (*p* .034) across forest management interventions. The mean aboveground biomass (1509.26 ton/ha), belowground biomass (301.85 ton/ha), carbon stock (851.22 ton/ha), and CO_2_ sequestration potential (3123.98 ton/ha) were all highest in the forest patches under area enclosure (Gubel, Dikuma, and Ambaser) (Table 6).

**TABLE 6:** Effects of forest management interventions on carbon stock (the values with different letters have significant differences according to mean separation).

Forest management intervention	Forest patches	AGB (ton/ha)	BGB (ton/ha)	TB (ton/ha)	Carbon (ton/ha)	CO_2_ (ton/ha)
Participatory forest management	Saharakani	1869.82	373.96	2243.78	1054.58	3870.30
	Den Maryam	1508.53	301.71	1810.24	850.81	3122.48
	Elala	1149.42	229.88	1379.31	648.27	2379.17
	Mean	1509.26^b^	301.85^b^	1811.11^b^	851.22^b^	3123.98^b^
Area exclosures	Ambaser	1161.40	232.28	1393.68	655.03	2403.96
	Gubel	2171.44	434.29	2605.73	1224.69	4494.62
	Dikuma	1951.35	390.27	2341.62	1100.56	4039.07
	Mean	1761.40^a^	352.28^a^	2113.68^a^	993.43^a^	3645.88^a^
Open forest	Darkan	798.36	159.67	958.04	450.28	1652.51
	Asech	690.66	138.13	828.79	389.53	1429.58
	Abuhay Dengara	698.30	139.66	837.96	393.84	1445.40
Mean		729.11^c^	145.82^c^	874.91^c^	411.21^c^	1509.17^c^
*p*‐value		.034				

### 3.4 Soil organic carbon across the forest patches

**FIGURE 3:**
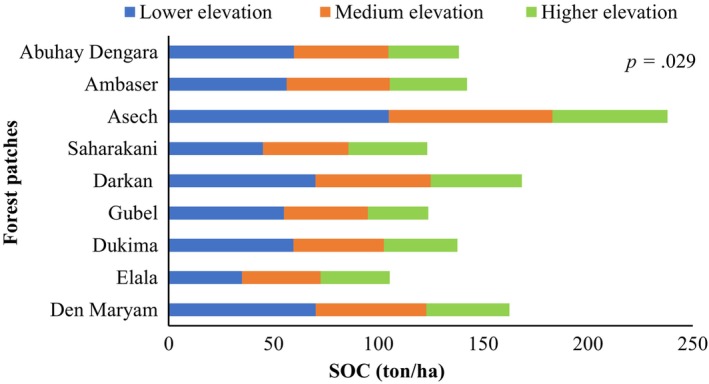
Soil organic carbon across forest patches and elevation gradients.

**FIGURE A1:**
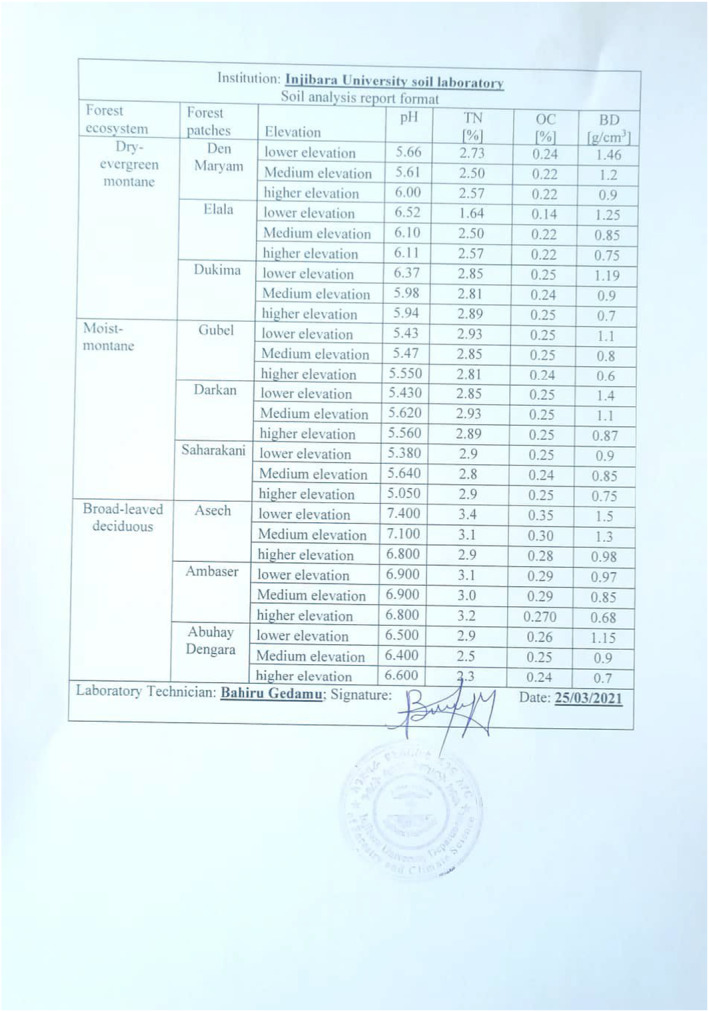
Soil result from laboratory.

Soil organic carbon (SOC) differed significantly (*p* .029) between forest patches and elevation gradient within forest patches. The highest SOC was found at lower elevation (mean: 61.7 ton/ha) and Asech forest patches (105 ton/ha), followed by the Darkan and Den Maryam forest patches (70 ton/ha) (Figure 3, Figure A1).

## 4 DISCUSSION

### 4.1 Carbon stock across environmental patterns

Because of participatory forest management interventions, Saharakani and Elala forest patches are relatively intact forests with large trees in both height and diameter. This may be the reason why these forest patches have a high carbon stock. The studied forest patches have the highest carbon stock as an overall pool (254–1177 ton/ha) than forest areas' carbon pools in other parts of Ethiopia (84.5–639.87 ton/ha) (Bazezew et al., 2015b; Belay et al., 2018; Feyissa et al., 2013; Gedefaw et al., 2013; Kendie et al., 2021; Solomon et al., 2017).

Furthermore, the studied forest ecosystems had the highest carbon stock potential among tropical, subtropical, and temperate natural forest ecosystems (Alvarez et al., 2012; Jindal et al., 2008; Macdicken et al., 2015; Malhi, 2010; Nizami, 2010; Payton & Weeks, 2012). Regardless of the circumstances, the study forest patches appear to be intact from the outside, yet they have degraded, resulting in a decrease in carbon stock.

The gentle slope has the greatest potential for biomass, carbon stock, and CO_2_ sequestration, all of which are directly related to vegetation growth performance. This is due to the gentle slope's ideal climatic and edaphic conditions for most woody species. The idea was supported by Yilma and Derero (2020) who found that environmental variables such as altitude, aspect, and slope have a greater influence on forest carbon dynamics. The mild slope provides the highest potential for biomass and carbon storage. The gentle slope is practically flat ground with stable soil and other biophysical factors that promote higher vegetation growth performance; this is why flat areas of forest patches have significant carbon reserves. The question is, why is the carbon stock on the medium slope smaller than on the high slope. Higher human and livestock pressures on the medium slope may lower the forest's value, whereas the steep slope is unsuitable for illegal harvesting and grazing. However, Dibaba et al. (2019) found that the carbon stock of woody species is negatively connected with slope patterns, meaning that carbon stock rises when the slope gradient lowers. This demonstrated that a moderate slope contains more carbon than a medium or steep slope. This concept is agreed upon and contradicts the study's findings. This gap could be explained by the strength of human and grazing stresses.

Total biomass, carbon stock, and potential CO_2_ sequestration have all reduced as altitude increased. This could be related to stem density, species diversity, and woody species' growth rates. The lower altitude, located at the forest's foot, has a high species diversity, suitable soil conditions, and a high stem density. The individual trees in flat slope are relatively tall with a great diameter. The tendency of all of the above trees' growth and edaphic conditions decline as altitude rise. This determines the carbon stock potential for the altitudinal patterns. This concept is consistent with Dibaba et al. (2019), who stated that carbon stock in woody species has a strong negative correlation with altitudinal gradients, indicating that carbon stock decreases as an altitude of a given forest increases. However, according to Feyissa et al. (2013), carbon stock overall has an increasing trend with an increasing altitudinal gradient. Scientifically, this study result is fairly acceptable because carbon stock is closely related to growth performance, vegetation features, and environmental factors. However, natural and anthropogenic disturbances may have an impact on whether the relationship between altitude and carbon stock is negatively or positively correlated.

Southwest‐facing has the highest carbon stock as a total carbon pool. Aspect parameters like as sunlight intensity and direction have a significant impact on vegetation growth performance and woody species diversity, both of which are linked to woody biomass and carbon stocks. In most studies by Alvarez et al. (2012), Feyissa et al. (2013), Assefa et al. (2013), Gedefaw et al. (2013), Liu et al. (2014), and Belay et al. (2018), north‐ and east‐facing countries with strong light intensity and perpendicular light direction, specifically in tropical countries, have higher vegetation performance and carbon stock. However, the studies suggest that this scenario is dependent on anthropogenic disturbances and forest management intensity. Most forest patches in this study are under grazing and human pressure (open forest patches without any management; Darkan, Den Maryam, and Asech), and some forests have hills in the south aspect that makes inaccessible for grazing and illegal harvesting (Dikuma and Saharakani forest patches). This could change the conventional wisdom that south‐facing is better for carbon storage. Woody species in the study forest, on the other hand, prefer south‐facing sites due to the need for a slight light concentration as a result of climate change and timberline shifts. Climate change, according to Ju and Turton (2014) and Payn et al. (2015) shifts the timberline in terms of altitude and aspect, causing most woody species in the tropics to prefer south and southwest facings.

### 4.2 Effects of forest management intervention on carbon stock

Some forest patches in the study area are managed through participatory forest management (Saharakani, Elala, and Abuhay Dengara), while others are subject to area exclosures. Forests under area exclosures management had enhanced carbon stock, which could be attributed to better woody species diversity, growth performance, and wood density of valuable important tree species. Forest management, as a general intervention, has the potential to raise forest value and consequently carbon stock capacity. In the study area, carbon stock increased by 41.4% from open, unmanaged forest to forest under area exclosures. There are numerous arguments that forest management interventions improve the carbon stock potential of forest ecosystems (Bazezew et al., 2015a, 2015b; Daba et al., 2022; Jati, 2012; Noormets et al., 2014). Furthermore, forest under area exclosures improves carbon stock potentials when compared to open forest patches, and it has intermediate biomass and carbon stock potential when compared to open forests and reference church forests (Kassaye et al., 2022). However, socioeconomic factors should be considered for more successful enhancement of natural forest biomass and carbon stock potential under area exclosures for long‐term climate change mitigation and adaptation (Kassaye et al., 2022).

### 4.3 Soil organic carbon across the forest patches

The study forest patches are located in various agroclimatic zones and alongside forest ecosystems, resulting in varying microclimatic conditions. The concentration of organic carbon is the most important determinant factor for soil organic carbon. Broad‐leaved deciduous forest ecosystems have higher soil organic carbon levels due to the high litterfall concentration and the rapid decomposition of it, whereas moist‐montane forest ecosystems have higher vegetation growth rates. The concept is similar to that of Dalle (2014), who stated that the broad‐leaved deciduous forest ecosystem found in lowland agroclimatic zones has the highest SOC due to its high decomposition rate, whereas the moist‐montane forest ecosystem has higher litter accumulation but a slow decomposition rate, thus having a low SOC.

Furthermore, the study by Lal (2008), Assefa et al. (2013), Alemu (2014), and Djibril et al. (2016) from around the world found that soil organic carbon is highly dynamic spatially and temporally, even on a small scale. It is a relatively long‐term carbon reservoir that plays an important role in climate change mitigation and adaption.

## 5 CONCLUSION

The forest patches located in moist‐montane forest ecosystems have the greatest potential for carbon storage, followed by the forest patches found in dry‐evergreen‐montane forest ecosystems and the forest patches found in broad‐leaved deciduous forest ecosystems. This is due to the high height and diameter growth of trees, varied environmental factors, and different forest ecosystems. Slope, aspect, and elevation gradients have all had a significant impact on woody biomass, carbon stock, and CO_2_ sequestration potential in the study area's natural forest patches. Overall, carbon stock and slope gradient are inversely related, meaning that carbon stock is higher on slight slopes. Carbon stock decreases proportionally to the elevation gradient. The southwest‐facing has the highest carbon stock. The aspect factor, which is influenced by sunlight intensity and direction, has a major impact on vegetation growth performance and woody species variety, both of which are linked to woody biomass and carbon stocks. The forest patches found in the Broad‐leaved forest ecosystem have greater soil organic carbon levels due to the quick decomposition of tree litter. The study area's natural forest patches have a large amount of carbon stock, which helps mitigate the crisis of climate change regardless of forest degradation or deforestation. The study concluded that forest management interventions, specifically forest management with area exclosures, can improve the carbon stock potential and values of forest ecosystems.

The study area's highest mean overall carbon pool (woody species and soil organic carbon) indicates that natural forests have a high potential for mitigating climate change by removing a reasonable amount of carbon from the atmosphere. However, appropriate conservation priorities and credit have yet to be assigned, and these resources are rapidly depleting. Even if there are some forest management interventions, it is not sure if area exclosures and PFM are successful or not (required further investigation). As a result, urgent conservation (area exclosures and PFM) for the open forest patches has been required, followed by appropriate and site‐specific sustainable forest management to maintain the health of the forest ecosystem (in terms of function, structures, and composition, particularly natural regeneration; number of seedlings > saplings > mature trees; and optimum species diversity, cover abundances, and lifeforms) for climate change mitigation, social stability, and economic benefit from natural forests in the long term. According to international commitments, international agencies such as REDD+ and the United Nations intergovernmental panel on climate change mitigation should be involved.

## AUTHOR CONTRIBUTIONS

**Melkamu Kassaye:** Conceptualization (equal); formal analysis (equal); investigation (equal); methodology (equal); software (equal); writing – original draft (equal). **Yonas Derebe:** Methodology (equal); supervision (equal); validation (equal); visualization (equal); writing – review and editing (equal). **Wondwossen Kibrie:** Methodology (equal); validation (equal); writing – review and editing (equal). **Fikadu Debebe:** Conceptualization (equal); data curation (equal); formal analysis (equal); writing – review and editing (equal). **Etsegenet Emiru:** Conceptualization (equal); methodology (equal); writing – review and editing (equal). **Bahiru Gedamu:** Data curation (equal); validation (equal); writing – review and editing (equal). **Mulugeta Tamir:** Conceptualization (equal); data curation (equal); supervision (equal); writing – review and editing (equal).

## CONFLICT OF INTEREST STATEMENT

There is no conflict of interest or copyright issue.

### OPEN RESEARCH BADGES

This article has earned an Open Data badge for making publicly available the digitally‐shareable data necessary to reproduce the reported results. The data is available at Mendeley data repository (https://doi.org/10.17632/7wwmwjgrzk.1).

## Data Availability

The datasets generated during and/or analyzed during the current study are available as supporting material uploaded in this journal submission portal. In addition, the data is available at: Mekonen (2024).

## APPENDIX A

**TABLE A1:** Carbon stock across the agroecology.

Carbon pool/forest community		*N*	Mean	SD	SE	95% confidence interval for mean		Minimum	Maximum
						Lower bound	Upper bound		
CO2 conc (ton/ha)	Highland	23	2288.1087	2835.54477	591.25196	1061.9272	3514.2902	14.80	10,603.20
	Mid‐altitude	36	1240.7722	1059.08345	176.51391	882.4299	1599.1145	18.30	3866.70
	Lowland	13	1089.0769	853.24657	236.64802	573.4652	1604.6887	193.60	2479.70
	Total	72	1547.9486	1852.35186	218.30176	1112.6673	1983.2300	14.80	10,603.20
Carbon stock (ton/ha)	Highland	23	623.4609	772.61992	161.10239	289.3550	957.5668	4.00	2889.10
	Mid‐altitude	36	338.0889	288.57841	48.09640	240.4480	435.7298	5.00	1053.60
	Lowland	13	296.7385	232.49792	64.48332	156.2414	437.2355	52.70	675.70
	Total	72	421.7833	504.72458	59.48236	303.1789	540.3878	4.00	2889.10
Total Biomass (ton/ha)	Highland	23	1326.5174	1643.88349	342.77340	615.6489	2037.3859	8.60	6147.10
	Mid‐altitude	36	719.3361	613.99784	102.33297	511.5891	927.0831	10.60	2241.70
	Lowland	13	631.3923	494.68866	137.20195	332.4549	930.3297	112.20	1437.60
	Total	72	897.4181	1073.88801	126.55892	645.0667	1149.7694	8.60	6147.10
Below‐ground Biomass (ton/ha)	Highland	23	264.7870	208.81435	43.54080	174.4889	355.0851	61.40	912.90
	Mid‐altitude	36	172.7472	214.82110	35.80352	100.0622	245.4322	13.70	989.90
	Lowland	13	113.1308	66.68605	18.49538	72.8328	153.4287	24.90	217.50
	Total	72	191.3847	200.13579	23.58623	144.3551	238.4143	13.70	989.90
Aboveground Biomass (ton/ha)	Highland	23	1323.9304	1044.06360	217.70231	872.4435	1775.4174	307.10	4564.50
	Mid‐altitude	36	863.7611	1074.10968	179.01828	500.3347	1227.1875	68.40	4949.70
	Lowland	13	565.6769	333.50012	92.49629	364.1448	767.2090	124.30	1087.50
	Total	72	956.9389	1000.67910	117.93116	721.7908	1192.0869	68.40	4949.70

**TABLE A2:** Carbon stock across the forest communities.

Carbon pool/forest community		*N*	Mean	SD	SE	95% confidence interval for mean		Minimum	Maximum
						Lower bound	Upper bound		
CO2 conc (ton/ha)	Darkan	8	288.6125	698.97277	247.12419	−295.7434	872.9684	14.80	2017.30
	Gubel	10	2396.0000	1231.73400	389.50849	1514.8706	3277.1294	721.80	4216.90
	Saharakani	5	5271.5200	4560.93336	2039.71141	−391.6268	10,934.6668	971.20	10,603.20
	Den Maryam	10	930.6000	917.49773	290.13826	274.2617	1586.9383	135.90	2917.10
	Dikuma	13	1912.6923	1140.18377	316.23008	1223.6861	2601.6985	177.30	3866.70
	Elala	13	807.4462	753.28621	208.92400	352.2399	1262.6525	18.30	2287.40
	Asech	5	1027.9400	836.53903	374.11163	−10.7604	2066.6404	440.60	2479.70
	Ambaser	3	333.6667	119.47424	68.97848	36.8762	630.4571	258.00	471.40
	Abuhay Dingara	5	1603.4600	844.84404	377.82574	554.4476	2652.4724	193.60	2249.00
	Total	72	1547.9486	1852.35186	218.30176	1112.6673	1983.2300	14.80	10,603.20
Carbon stock (ton/ha)	Darkan	8	78.6375	190.46744	67.34041	−80.5973	237.8723	4.00	549.70
	Gubel	10	652.8700	335.61813	106.13177	412.7833	892.9567	196.70	1149.00
	Saharakani	5	1436.3600	1242.74686	555.77329	−106.7140	2979.4340	264.60	2889.10
	Den Maryam	10	253.5600	249.98029	79.05071	74.7349	432.3851	37.00	794.80
	Dikuma	13	521.1769	310.67638	86.16612	333.4371	708.9168	48.30	1053.60
	Elala	13	220.0231	205.26856	56.93126	95.9805	344.0656	5.00	623.30
	Asech	5	280.1000	227.95426	101.94424	−2.9426	563.1426	120.00	675.70
	Ambaser	3	90.9000	32.52860	18.78040	10.0945	171.7055	70.30	128.40
	Abuhay Dingara	5	436.8800	230.21930	102.95720	151.0250	722.7350	52.70	612.80
	Total	72	421.7833	504.72458	59.48236	303.1789	540.3878	4.00	2889.10
Total Biomass (ton/ha)	Darkan	8	167.3250	405.21598	143.26548	−171.4440	506.0940	8.60	1169.50
	Gubel	10	1389.0700	714.09566	225.81687	878.2367	1899.9033	418.50	2444.70
	Saharakani	5	3056.1200	2644.16342	1182.50583	−227.0425	6339.2825	563.00	6147.10
	Den Maryam	10	539.5200	531.91568	168.20651	159.0104	920.0296	78.80	1691.20
	Dikuma	13	1108.8769	661.01381	183.33225	709.4303	1508.3236	102.80	2241.70
	Elala	13	468.1154	436.71580	121.12317	204.2107	732.0201	10.60	1326.10
	Asech	5	595.9400	484.98998	216.89411	−6.2546	1198.1346	255.40	1437.60
	Ambaser	3	193.4333	69.27643	39.99676	21.3411	365.5255	149.60	273.30
	Abuhay Dingara	5	929.6200	489.83048	219.05885	321.4151	1537.8249	112.20	1303.90
	Total	72	897.4181	1073.88801	126.55892	645.0667	1149.7694	8.60	6147.10
Below‐ground Biomass (tone/ha)	Darkan	8	159.6625	70.75539	25.01581	100.5095	218.8155	61.40	280.80
	Gubel	10	294.3000	200.02215	63.25256	151.2128	437.3872	94.00	666.20
	Saharakani	5	373.9600	319.15447	142.73022	−22.3226	770.2426	127.80	912.90
	Den Maryam	10	101.7100	96.96194	30.66206	32.3476	171.0724	13.70	313.30
	Dikuma	13	170.2615	140.78883	39.04780	85.1837	255.3394	17.60	468.30
	Elala	13	229.8769	316.19332	87.69625	38.8032	420.9506	15.90	989.90
	Asech	5	138.1200	18.92121	8.46182	114.6262	161.6138	105.40	153.00
	Ambaser	3	27.3000	2.08806	1.20554	22.1130	32.4870	24.90	28.70
	Abuhay Dingara	5	139.6400	76.13608	34.04909	45.1046	234.1754	55.60	217.50
	Total	72	191.3847	200.13579	23.58623	144.3551	238.4143	13.70	989.90
Aboveground Biomass (ton/ha)	Darkan	8	798.3625	353.77333	125.07776	502.6006	1094.1244	307.10	1404.10
	Gubel	10	1471.4400	1000.13388	316.27010	755.9873	2186.8927	469.80	3331.20
	Saharakani	5	1869.8200	1595.75010	713.64114	−111.5655	3851.2055	639.20	4564.50
	Den Maryam	10	508.5300	484.79421	153.30539	161.7291	855.3309	68.40	1566.30
	Dikuma	13	851.3538	703.90909	195.22925	425.9858	1276.7219	88.00	2341.40
	Elala	13	1149.4231	1580.98315	438.48583	194.0445	2104.8016	79.60	4949.70
	Asech	5	690.6600	94.53980	42.27948	573.2733	808.0467	527.20	765.00
	Ambaser	3	136.3333	10.46438	6.04161	110.3384	162.3283	124.30	143.30
	Abuhay Dingara	5	698.3000	380.67106	170.24127	225.6345	1170.9655	278.10	1087.50
	Total	72	956.9389	1000.67910	117.93116	721.7908	1192.0869	68.40	4949.70

**TABLE A3:** Carbon stock across the slope pattern.

Carbon pool/forest community		*N*	Mean	Std. deviation	Std. error	95% confidence interval for mean		Minimum	Maximum
						Lower bound	Upper bound		
CO2 conc (ton/ha)	Lower	26	2186.4808	2539.68144	498.07251	1160.6812	3212.2803	18.30	10,603.20
	Medium	21	1490.1476	1127.44379	246.02840	976.9414	2003.3539	14.80	3866.70
	Higher	25	932.4280	1238.29492	247.65898	421.2850	1443.5710	17.70	4216.90
	Total	72	1547.9486	1852.35186	218.30176	1112.6673	1983.2300	14.80	10,603.20
Carbon stock (ton/ha)	Lower	26	595.7731	692.00075	135.71251	316.2679	875.2782	5.00	2889.10
	Medium	21	406.0286	307.20266	67.03712	266.1916	545.8655	4.00	1053.60
	Higher	25	254.0680	337.41837	67.48367	114.7885	393.3475	4.80	1149.00
	Total	72	421.7833	504.72458	59.48236	303.1789	540.3878	4.00	2889.10
Total Biomass (ton/ha)	Lower	26	1267.5962	1472.35959	288.75347	672.8972	1862.2951	10.60	6147.10
	Medium	21	863.9190	653.63158	142.63410	566.3895	1161.4486	8.60	2241.70
	Higher	25	540.5720	717.89894	143.57979	244.2379	836.9061	10.30	2444.70
	Total	72	897.4181	1073.88801	126.55892	645.0667	1149.7694	8.60	6147.10
Below‐ground Biomass (tone/ha)	Lower	26	256.3615	274.92939	53.91809	145.3152	367.4079	13.70	989.90
	Medium	21	156.3000	107.17110	23.38665	107.5163	205.0837	17.60	468.30
	Higher	25	153.2800	151.73579	30.34716	90.6465	215.9135	24.00	666.20
	Total	72	191.3847	200.13579	23.58623	144.3551	238.4143	13.70	989.90
Aboveground Biomass (ton/ha)	Lower	26	1281.8115	1374.65406	269.59184	726.5767	1837.0463	68.40	4949.70
	Medium	21	781.5238	535.87001	116.93642	537.5987	1025.4489	88.00	2341.40
	Higher	25	766.4200	758.66584	151.73317	453.2581	1079.5819	120.20	3331.20
	Total	72	956.9389	1000.67910	117.93116	721.7908	1192.0869	68.40	4949.70

**TABLE A4:** Carbon stock inferential statistics.

Tests of between‐subjects effects						
Source	Dependent variable	Type III sum of squares	df	Mean square	*F*	Sig.
Corrected Model	Agroecology	34.611	15	2.307		
	Aboveground Biomass (ton/ha)	20,915,299.408	15	1,394,353.294	1.556	.117
	Belowground Biomass (tone/ha)	836,642.994	15	55,776.200	1.556	.117
	Total Biomass (ton/ha)	44,950,874.694	15	2,996,724.980	4.544	.000
	Carbon stock (ton/ha)	9,929,712.050	15	661,980.803	4.544	.000
	CO2 conc (ton/ha)	133,743,156.168	15	8,916,210.411	4.544	.000
Intercept	Agroecology	187.703	1	187.703		
	Aboveground Biomass (ton/ha)	39,307,591.357	1	39,307,591.357	43.866	.000
	Belowground Biomass (tone/ha)	1,572,290.270	1	1,572,290.270	43.866	.000
	Total Biomass (ton/ha)	51,008,654.948	1	51,008,654.948	77.351	.000
	Carbon stock (ton/ha)	11,267,560.094	1	11,267,560.094	77.352	.000
	CO2 conc (ton/ha)	151,764,378.978	1	151,764,378.978	77.351	.000
Forest	Agroecology	33.080	8	4.135		
	Aboveground Biomass (ton/ha)	12,446,156.105	8	1,555,769.513	1.736	.110
	Belowground Biomass (tone/ha)	497,870.656	8	62,233.832	1.736	.110
	Total Biomass (ton/ha)	32,811,086.109	8	4,101,385.764	6.219	.000
	Carbon stock (ton/ha)	7,247,894.071	8	905,986.759	6.220	.000
	CO2 conc (ton/ha)	97,623,026.707	8	12,202,878.338	6.220	.000
Alt	Agroecology	0.000	2	0.000		
	Aboveground Biomass (ton/ha)	119,279.047	2	59,639.523	.067	.936
	Belowground Biomass (tone/ha)	4773.288	2	2386.644	.067	.936
	Total Biomass (ton/ha)	2,489,289.139	2	1,244,644.570	1.887	.161
	Carbon stock (ton/ha)	549,914.452	2	274,957.226	1.888	.161
	CO2 conc (ton/ha)	7,406,590.767	2	3,703,295.383	1.888	.161
Slope	Agroecology	0.000	2	0.000		
	Aboveground Biomass (ton/ha)	4,225,450.559	2	2,112,725.280	2.358	.104
	Belowground Biomass (tone/ha)	169,046.861	2	84,523.431	2.358	.104
	Total Biomass (ton/ha)	4,934,542.118	2	2,467,271.059	3.741	.030
	Carbon stock (ton/ha)	1,090,144.887	2	545,072.443	3.742	.030
	CO2 conc (ton/ha)	14,682,311.542	2	7,341,155.771	3.742	.030
Aspect	Agroecology	0.000	3	0.000		
	Aboveground Biomass (ton/ha)	4,799,868.922	3	1,599,956.307	1.785	.160
	Belowground Biomass (tone/ha)	192,003.381	3	64,001.127	1.786	.160
	Total Biomass (ton/ha)	4,157,112.256	3	1,385,704.085	2.101	.110
	Carbon stock (ton/ha)	918,358.279	3	306,119.426	2.102	.110
	CO2 conc (ton/ha)	12,368,616.373	3	4,122,872.124	2.101	.110
Error	Agroecology	0.000	56	0.000		
	Aboveground Biomass (ton/ha)	50,181,164.924	56	896,092.231		
	Belowground Biomass (tone/ha)	2,007,214.659	56	35,843.119		
	Total Biomass (ton/ha)	36,928,843.232	56	659,443.629		
	Carbon stock (ton/ha)	8,157,317.850	56	145,666.390		
	CO2 conc (ton/ha)	109,872,569.192	56	1,962,010.164		
Total	Agroecology	284.000	72			
	Aboveground Biomass (ton/ha)	137,029,171.000	72			
	Belowground Biomass (tone/ha)	5,481,081.710	72			
	Total Biomass (ton/ha)	139,865,577.910	72			
	Carbon stock (ton/ha)	30,895,914.880	72			
	CO2 conc (ton/ha)	416,138,158.350	72			
Corrected Total	Agroecology	34.611	71			
	Aboveground Biomass (ton/ha)	71,096,464.331	71			
	Belowground Biomass (tone/ha)	2,843,857.653	71			
	Total Biomass (ton/ha)	81,879,717.927	71			
	Carbon stock (ton/ha)	18,087,029.900	71			
	CO2 conc (ton/ha)	243,615,725.360	71			
